# Interactions between perceived stress and microbial-host immune components: two demographically and geographically distinct pregnancy cohorts

**DOI:** 10.1038/s41398-022-02276-3

**Published:** 2023-01-06

**Authors:** Beatriz Peñalver Bernabé, Pauline M. Maki, Janet L. Cunningham, Tory Eisenlohr-Moul, Lisa Tussing-Humphreys, Ian M. Carroll, Samantha Meltzer-Brody, Jack A. Gilbert, Mary Kimmel

**Affiliations:** 1 Department of Biomedical Engineering, College of Engineering and Medicine, University of Illinois Chicago, Chicago, IL USA; 2 Center of Bioinformatics and Quantitative Biology, University of Illinois Chicago, Chicago, IL USA; 3 Department of Psychology, College of Medicine, University of Illinois Chicago, Chicago, IL USA; 4 Department of Psychiatry, College of Medicine, University of Illinois Chicago, Chicago, IL USA; 5 Department of Obstetrics and Gynecology, College of Medicine, University of Illinois Chicago, Chicago, IL USA; 6 Department of Medical Sciences, Psychiatry, Uppsala University, Uppsala, Sweden; 7 Department of Kinesiology and Nutrition, College of Applied Health Sciences, University of Illinois Chicago, Chicago, IL USA; 8 Department of Nutrition, School of Public Health, University of North Carolina, Chapel Hill, NC USA; 9 Department of Psychiatry, University of North Carolina, Chapel Hill, NC USA; 10 Department of Pediatrics and Scripps Institution of Oceanography, University of California San Diego, La Jolla, CA USA; 11 Department of Women’s and Children’s Health, Uppsala University, Uppsala, Sweden

**Keywords:** Predictive markers, Genomics

## Abstract

Higher stress during pregnancy associates with negative outcomes and elevated inflammation. The gut microbiota, reflecting environment and social interactions, alongside host immune responses have the potential to better understand perceived stress and identify when stress is excessive in pregnancy. Two U.S. cohorts of 84 pregnant individuals, composed of urban women of color and suburban white women, completed the Perceived Stress Scale-10 (PSS-10) and provided fecal and blood samples at two time points. Confirmatory Factor Analysis assessed the robustness of a two-factor PSS-10 model (Emotional Distress/ED and Self-Efficacy/SE). Gut microbiota composition was measured by 16 S rRNA amplicon sequencing and the immune system activity was assessed with a panel of 21 T-cell related cytokines and chemokines. ED levels were higher in the suburban compared to the urban cohort, but levels of SE were similar. ED and SE levels were associated with distinct taxonomical signatures and the gut microbiota data improved the prediction of SE levels compared with models based on socio-demographic characteristics alone. Integration of self-reported symptoms, microbial and immune information revealed a possible mediation effect of *Bacteroides uniformis* between the immune system (through CXCL11) and SE. The study identified links between distinct taxonomical and immunological signatures with perceived stress. The data are congruent with a model where gut microbiome and immune factors, both impacting and reflecting factors such as close social relationships and dietary fiber, may modulate neural plasticity resulting in increased SE during pregnancy. The predictive value of these peripheral markers merit further study.

## Introduction

Pregnancy is a time of biological, social and psychological adaptation [1, 2]. High Perceived Stress (PS), defined by Cohen as nervousness, fear, and anger resulting from perceiving that one’s life is out of control with troubles that one can’t overcome [3]; increases the risk for development of anxiety and depressed mood [4–6]. High PS during pregnancy has also been associated with obstetrical complications, such as gestational diabetes mellitus, preterm birth, gestational hypertension, preeclampsia and complications for the infant, such as being small for gestational age [7–11].

The way that stress is experienced and expressed, and the resulting physical and mental health effects, is highly individualized; and may be influenced by factors such as culture. The data-driven Woods-Giscombe’s Superwoman schema explains that Black women feel an obligation to manifest strength and independence, suppress emotions, not express feelings of stress, and succeed despite limited resources; all while helping others [12]. Even despite on average reporting lower levels of PS, structural inequities and chronic experienced stressors may explain poorer health outcomes including Black women and Latinas being at much higher risk of obstetrical complications (e.g., preterm birth, gestational diabetes mellitus, pre-eclampsia) [12–14]. In contrast, a study of U.S. non-pregnant individuals with over 56% female who self-identified as Mexican-origin, participants that reported higher PS had lower 10-year cardiovascular disease risk [15]. This indicates that self-reported PS is a complex construct that results from the balance of the individual’s emotional sense of distress (perceived helplessness, perceived distress) and their coping mechanisms (perceived self-efficacy, perceived coping) and that could be impacted by cultural and environmental factors. Thus, it is important to assess for everyone how distress may be balanced by motivation to harness supports and sense of coping and not only total perceived stress as the balance of distress and coping is dependent of the population [16, 17]. Furthermore, report of perceived stress may differ due to pressures to disclosure coping abilities and strength against the varied consequences for different groups [16, 17].

Biological markers that accurately reflect stress levels and negative health effects could serve to further understand beyond self-report this complex balance of distress and coping, and to identify those individuals at risk for negative health outcomes [18–20]. There is increasing evidence that the microbiota-gut-brain axis, encompassing interactions between the gut microbes with neurological, endocrinological and immunological systems, is associated with mental health disorders and obstetrical complications [10, 21–26]. Disruptions in the gut microbial ecosystem have been associated with both intestinal and systemic diseases (e.g., IBS, diabetes, depression) [27]. Initial cross sectional studies in pregnancy indicate an association between gut microbial communities, maternal psychosocial stress, and host immune system responses [28, 29]. The host immune system is changing during pregnancy and is modulated by depression and anxiety. For instance, Osborne, et. al. found an increase in pro-inflammatory innate immunity cytokines in the third trimester in individuals with higher depression and anxiety [30]. The integration of gut microbiome and markers of the immune system, such as circulating cytokines and chemokines, could be potential ideal biological biomarkers of PS, emotional distress and coping (self-efficacy) and the balance between them. Furthermore, the gut microbiota reflect lifestyle (e.g., diet, exercise), residential location and home environment (e.g., rural versus urban), medication use (e.g., antibiotics) [31], and close social interactions [32] creating biomarkers able to reveal some of these factors more succinctly.

We recruited a total of 84 pregnant individuals from two sociodemographic and geographically distinct U.S. locations: from an urban population, the majority women of color, in the Midwest and from a suburban population, the majority White, from the US south. We aimed to understand the maternal gut-microbiota-brain axis during the second and third trimesters in relation to PS through integration of the maternal gut microbiota composition and maternal cytokine and chemokine concentrations in serum. We hypothesized that models that included maternal gut microbiota and systemic inflammatory characteristics will better predict PS levels during pregnancy than those models based on socio-demographic characteristics alone.

## Materials and methods

### Participant recruitment

Our sample included US pregnant individuals from two sociodemographic and geographically distinct cohorts (self-reported): urban-dwelling women of color (Black women and Latinas) with low socioeconomic status (SES) and low formal education from a Midwest large city, Chicago (Illinois); suburban White women with higher SES and higher level of formal education from a southern city, Chapel Hill (North Carolina). Urban participants were recruited during their initial obstetric visit at a public university hospital before 16 weeks of gestation. Suburban participants were recruited through advertisement and their initial visit was before 28 weeks of gestation. Participants provided fecal samples during the second trimester (T2); and the third trimester (T3) and blood samples at the T2 visit. Study protocols were approved by the University of Illinois Chicago (IRB# 2014-0325, IRB# 2018-0842) and the University of North Carolina (IRB# 16-0959, IRB# 16-2783) Institutional Review Boards. All individuals were consented before engaging in the study at the respective sites. In the urban cohort, individuals were excluded if they took antibiotics 6 months before the beginning of the study. Individuals in the suburban cohort were not excluded based on antibiotics, but a thorough medication history was taken at each visit (three at the first visit were taking an oral antibiotic, one at visit 2, five at visit 4; the individual who took an antibiotic in visit 2 also took one in visit 4 and was the only subject to take and antibiotic at more than one time point). Given small numbers antibiotic use was not included in analyses.

### Self-reported mental health questionnaires

At each visit, participants completed questionnaires about demographics (e.g., race, ethnicity, education) and the self-assessment tools including the Generalized Anxiety Disorder-7 (GAD-7) [33], Perceived Stress Scale (PSS)-10 [3, 34], and either the Patient Health Questionnaire (PHQ-9) [35, 36] in the urban or the Edinburgh Postnatal Depression Scale (EPDS) [37] in the suburban cohorts. Participants also consented to access their electronic medical records. BMI was measured during the participant’s routine obstetric visit (urban cohort) or at the research visit (suburban cohort). The PSS-10 is one of the most common self-report tools used to assess PS [3, 34], comprised of ten items [3] that are scored on a five-point Likert scale ranging from 0 “*Never*” to 4 “*Very often*.”

### Confirmatory factor analysis (CFA)

In both non-pregnant and pregnant individuals, PSS-10 has been consistently divided into two factors, “perceived helplessness” or “perceived distress” and “perceived self-efficacy” [17] or “perceived coping” [38, 39]. Perceived distress (Emotional Distress, ED) and perceived Self-Efficacy (SE) provide information about the balance between perceived distress and perceived resilience—characterized by a ratio between ED and SE. CFA using latent variable modeling implemented in the R package *lavaan* [40] was employed to determine adequacy of the two-latent variable model of PSS-10 and its configural, metric (loading), scalar (intercept) and residual invariance between the two populations. Models with comparative fit index and Tucker-Lewis Index > 0.95 and root mean square error of approximation (rmsea) < 0.05 were deemed appropriate for fitting the data. Nested models were compared with ANOVA using a statistically significant *p* value cutoff < 0.05. (Supplemental methods).

### Reduced PSS-10

To identify the core PSS-10 items that support model structural invariance between the two cohorts, we employed an iterative process by removing one PSS-10 item at a time until the model metric, scalar and residual invariabilities remained invariant. Using the Lagrange Multiplier test, the PSS-10 item with the lowest *p* value was removed from the model until the ANOVA comparisons between the nested models was no longer statistically significant (*p* > 0.05). Analyses were performed using the “*lavaan*” package [40]. Reduced factors are denoted with a “r”. All analyses included each factor ED, SE, along with the total PS and the ratio between ED and SE. Reduced PSS factors and the total reduced PS are noted as rED, rSE, rPS, respectively. These was necessary to ensure comparison across the two cohorts as indicated above.

### Exploratory factor analysis

Correlations between the PSS-10 Likert scores were calculated using Polychoric correlation with an oblique rotation, “Promax”, as PSS-10 Likert scores are highly correlated. The number of dimensions was selected using paralleled analysis with maximum likelihood as factoring method. Loadings for each question were estimated using minimum residuals with 2000 iterations. Internal consistency between the items assigned in each symptom dimension was determined with Cronbach’s alpha [41]. Analyses were performed using the “*psych*” package [42].

### Cytokine analysis

Blood samples were collected by a trained phlebotomist during a clinical or research visit, processed for serum, aliquoted and stored at −80 °C and analyzed in duplicate at the University of Chicago (Chicago, IL, US) using a 21 T-cell specific panel: CX3CL1, GM-CSF, IFNγ, IL-1β, IL-2, IL-4, IL-5, IL-6, IL-7, IL-8, IL-10, IL-12 (p70), IL-13, IL-17A, IL-21, IL-23, CXCL11, CCL3, CCL4, CCL20, TNF-α (HSTCMAG28SPMX21 from Milliplex®). We assessed cytokine and chemokine levels in serum in the second trimester for a subset of participants (*n* = 28).

### Fecal sample collection

Most participants from the urban cohort provided rectal swabs (96%) apart from three samples from three different participants that provided stool. Samples were processed and stored at −80 °C within 2 h of collection. Stool samples from the suburban cohort were collected at home. Individuals were provided a stool kit and were instructed to add fecal matter into tube that contained a solvent to preserve the DNA and RNA in the samples at room temperature (DNA/RNA Shield, Zymo Research, Irvine, CA). Samples were then placed in a Styrofoam cooler with an ice pack and collected by the study team within 48 h (encouraged to collect within 24 h). Stool specimens from both locations were homogenized, aliquoted, and then frozen at −80 °C.

### Fecal DNA extraction and sequencing

DNA was extracted with QIAGEN® MagAttract® PowerSoil® DNA KF Kit [43], as previously described [44]. The V4 region of the 16 S rRNA gene of the barcoded samples was targeted with 515F-806R. Samples were sequenced on a 151 bp × 12 bp × 151 bp MiSeq run. Empty blanks were included to control for external and cross-contamination: (i) clean swabs to identify possible contamination within them, *n* = 16; (ii) open rectal swabs exposed to the biosafety cabinet environment during the extraction and barcoding protocol, *n* = 16; (iii) empty vials for contamination in the extraction kits and downstream processing, *n* = 35.

### Sequence identification and filtering

Amplicon Sequence Variants (ASV) were determined with *DADA2*, using default parameters [45]. Paired reverse and forward sequences were filtered and truncated to 150 base-pair length with maximum expectation of 0.75. Out of the 186 samples, ten samples were removed due to low number of reads (<5): five were controls, two from the urban, three from the suburban. After inference and chimeric removal, only sequences whose lengths were between 251 and 254 bp were used for downstream analysis. Taxonomy was assigned using the Silva database v.132 (ref. [46]). ASVs coming from contamination were removed. We assumed that ASVs that were not present in at least 90% of the samples (excluding controls) were contaminants. Samples whose relative abundance was <1% and all their reads were below 10 counts were also removed.

### Cytokine and chemokines analysis

Association between serum cytokines and chemokines concentrations, in logarithmic scale, were estimated using linear models thar were adjusted by participant’s BMI and recruitment site. BMI was obtained either by participant’s electronical medical records (urban) or by researchers (suburban). Clustering was performed with k-means. Correlations between cytokines and chemokines concentrations, in logarithmic scale, with perceived stress dimensions (ED, SE, and PS) was determined with Spearman correlations and using linear models corrected for participant’s BMI.

### Gut microbiome analysis

Counts were normalized using cumulative sum scaling normalization (CSS) [47]. Alpha- and beta-diversity were calculated with *phyloseq* [48]. For alpha-diversity, we employed total observed ASV and Chao to estimate richness and Shannon, Inverse Simpson and Abundance-based Coverage Estimator to estimate evenness. Beta-diversity volatility between T2 and T3 was calculated by Bray–Curtis and by normalized UNIFRAC distances. Distances calculated with UNIFRAC [49] were not based on samples rarefied to 6000 counts. Statistically significant differences in alpha- and beta-diversity with respect to PS dimensions (ED, SE, and PS) were determined with PERMANOVA, adjusting by trimesters and cohort. Volatility differences as a function of gestational weeks differences between trimesters were assessed with linear models correcting by cohort. Associations between PS dimensions (ED, SE, PS, and PS Ratio) and ASVs were calculated using zero-inflated Generalized Linear Models adjusted by cohort and participant’s age and the log2 CSS normalization factors for each sample using *metagenomeSeq* [50]. For serum cytokines and chemokines abundance, models were corrected by participants’ BMI, age and cohort. Unless stated otherwise, all *p* values were corrected by multiple comparisons using false discovery rate (fdr).

### Linear mixed models (LMM)

Following Rothschild et al., we employed LMM to identify the predictability power of the gut microbiome (microbiome association index) to determine PS [51]. Normalized ASV counts were summarized at the genus levels to construct the taxa kinship matrix. LMM for all reduced PSS-10 dimensions were corrected by statistically significant covariates that were determined using a stepwise approach. Except for rSE scores that were corrected by nulliparity, rPS and rED were corrected by race. LMMs with and without the microbiota kinship matrix, using participants as random variables were compared and validated using a ten-times cross-validation approach (Supplemental Methods).

### Co-abundances networks

Correlations between CSS normalized genera were obtained with SparcCC [52]. Edge significance was determined with 100 bootstrapping iterations (null-model with scrambling the count data). Edges were deemed significant if their correlation coefficient and their *z*-scores were three times greater than the standard deviations of the corresponding bootstrapping correlations and *z*-scores for the sample edge.

### Statistical analysis

Unless otherwise specified, chi-square tests were used for comparisons of categorical variables, *t*-tests for categorial and continuous variables, and Spearman partial and non-partial correlations for continuous variables. All the *p* values were corrected for multiple comparisons using fdr [53]. Analysis was conducted in R (ref. [54]) and figures were produced with *ggplot2* [55].

## Results

### Emotional distress (ED) and self-efficacy (SE) factors measured different concepts of perceived stress (PS) in the urban and suburban cohorts

The urban cohort (*n* = 38) comprised more than 60% Black women and ~31% Hispanic women or Latinas. The suburban cohort (*n* = 46) comprised 78% non-Hispanic White women. Among the suburban cohort, 61% reported an education status of college or above, 96% were married or in committed relationship, and 28% were obese. This compared to women in the urban cohort in which only 16% completed some college education, 60% were married or in a committed relationship, more than 44% were obese and were slightly younger than the suburban group (Table 1).

**Table 1:** Demographic characteristics of the urban and suburban cohorts.

	Urban	Suburban	Urban	Suburban
	*N* = 38	*N* = 46		%
Hispanic Non-Black*	12.0	1.0	31.6	2.2
Non-Hispanic Black**	24.0	5.0	63.2	10.9
Non-Hispanic White**	3	36	7.9	78.3
Committed Relationship**	23	44	60.5	95.7
Bachelor or above**	6	28	15.8	60.9
Nulliparous	12	19	31.6	41.3
Obese	17	13	44.7	28.3
Gestational Diabetes	0	2	0.0	4.3
Pre-eclampsia	0	2	0.0	4.3
	**Average**		**StDev**	
Age**	28.6	32.3	5.5	3.0
BMI	32.0	28.9	8.2	6.0

N number of participants, *BMI* body mass index, false discovery rate corrected *p* values. Gestational weeks at the T2 [24, 29] and at the T3 [33, 38] visits.

**p < 0.001; *p < 0.01.

While PSS-10 could be explained by a two factor model as previously reported [17] (Fig. S1a, Table S1), the confirmatory factor analysis (CFA) indicated that the loading of the PSS-10 items in each factors, ED and SE, were very distinct between the two cohorts (Table 2, Fig. S1b, c). Using an iterative step approach, we removed an item at a time to arrive to a significant reduced PSS-10 with seven total items. The strict two-factor model of the reduced PSS-10 was significant (Fig. S2a, Table S2) and was strictly invariant between the urban and suburban cohort (Table 3). The items removed from PSS-10 to render an invariance model were *item f* from the ED factor (‘*In the last month, how often have you found that you could not cope with all the things that you had to do?*‘) and *items e* and *h* from the SE factor (‘*In the last month, how often have you felt that things were going your way?*’; ‘*In the last month, how often have you felt that you were on top of things?*’). Levels of the rPS and the rED differed by trimesters and location, with suburban women reporting higher levels of rPS and of rED than the urban women in the third trimester and overall, but we didn’t observe any differences in rSE between the cohorts or trimesters (Table 4).

**Table 2:** Confirmatory factor analysis results.

	Df	Chisq	Chisq.diff	Df.diff	*p* value
Configural	68	61.428			
Weak	76	99.694	24.187	8	0.002
Weak	76	99.693			
Strong	84	92.639	−13.648	8	1.000
Strong	84	92.639			
Strict	94	146.242	41.053	10	<0.001

The CFA established that the two-factor model of PS is not invariant and depends on the cohort. *Df* degrees of freedom, *Chisq* chi-square values, *Chisq.diff* differences between the chi-square values for the two models, *Df. diff* difference between the degrees of freedom; configural model, latent model with two factors without any constrain; weak model, constraining the loads of each item in each factor to be equal between groups; strong, constraining the loads and the intercepts for each item in each factor to be equal between groups; strict model, constraining the loads, the intercepts and residuals of the latent model for each item in each factor to be equal between groups.

**Table 3:** CFA results of reduced PSS-10 questionnaire confirmed that the reduced two factor model of PS is invariant between cohorts.

	Df	Chisq	Chisq.diff	Df.diff	*p* value
Configural	26	18.381			
Weak	31	23.177	4.008	5	0.548
Weak	31	23.177			
Strong	36	27.423	8.898	5	0.113
Strong	36	27.423			
Strict	43	36.934	10.891	7	0.143

**Table 4:** PS, ED and SE levels in the two sites.

	T2 and T3 combined				T2				T3			
	Urban	Suburban	Urban	Suburban	Urban	Suburban	Urban	Suburban	Urban	Suburban	Urban	Suburban
	*N*		%		*N*		%		*N*		%	
MDD (PHQ-9 > 9 or EPDS > 10)	4	6	10.5	13	2	4	8.0	16.0	3	6	7.1	14.2
GAD-7 > 9	4	6	10.5	13	4	1	16.0	4.0	3	5	7.1	11.9
PSS-10 > 13*	14	26	36.8	56.5	11	7	44.0	28.0	7	25	16.7	59.2
MDD & GAD7	3	4	7.9	8.7	1	1	4.0	4.0	3	3	7.1	7.1
MDD & PSS	3	6	7.9	13	1	2	4.0	8.0	2	6	4.7	14.2
MDD & GAD7 & PSS	2	4	5.3	8.7	0	1	0.0	4.0	2	3	4.7	7.1
GAD7 & PSS	3	6	7.9	13	3	1	12.0	4.0	2	5	4.7	11.9
	**Average**		**StDev**		**Average**		**StDev**		**Average**		**StDev**	
GAD-7	2.7	3.8	3.7	3.9	2.7	2.9	3.8	3.3	2.6	4.4	3.7	4.2
PSS-10*	10.3	14.4	7.6	6.5	11.5	12.8	7.3	6.6	9.1	15.3	7.9	6.2
Emotional Distress**	4.8	9.2	5	4.2	5.4	8.2	5.2	4.4	4.1	9.7	4.8	4.0
Self-Efficacy	10.5	10.8	4.7	2.8	10.0	11.4	5.0	2.7	11.0	10.4	4.4	2.8
PSS-10 (reduced)**	6.6	10.2	5.4	4.5	7.5	9.1	5.3	4.7	5.7	10.8	5.5	4.3
Emotional Distress (reduced)**	4.1	7.8	4.3	3.4	4.7	7.1	4.5	3.6	3.5	8.2	4.1	3.3
Self-Efficacy (reduced)	5.4	5.6	2.5	1.5	5.1	6.0	2.7	1.5	5.8	5.3	2.3	1.5

While there were several statistically significant differences in total PS and ED between the cohorts, we didn’t identify any statistically significant differences between self-reported levels of SE or anxiety as measured by GAD-7. We didn’t identify any statistically significant differences in the percentage of participants that self-reported moderate/severe depression symptoms based on EPDS ≥ 12 (suburban) or PHQ-9 > 9 (urban).

*N* number of participants, *MDD* Major Depression Disorder, *PHQ-9* Patient Health Questionnaire 9-items, *EPDS* Edinburg Postpartum Depression Score, *GAD-7* Generalized Anxiety Disorder 7-item.

**p < 0.001, *p < 0.01

**Fig. 1:**
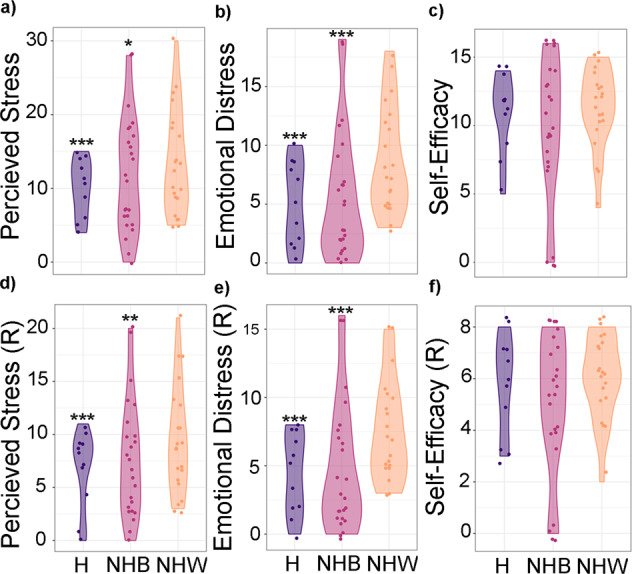
Total PS (PSS-10), ED and SE scores, including the reduced form of PSS-10 per race and ethnicity. While the levels of Total PS and ED (**a**, **b**) as well as their reduced forms (**d**, **e**) were higher in non-Hispanic White individuals, we observed no statically significant differences in SE (**c**) nor in the rSE values (**f**). R reduced, H Hispanic/Latina, NHB Non-Hispanic Black, NWH Non-Hispanic White; *p < 0.05; **p < 0.01; ***p < 0.001.

Participants from the urban cohort reported lower levels of PS and ED (PSS-10 subconstruct) (Table 4). In addition, the ratio ED and SE showed differences between the two cohorts with the urban participants reporting lower levels of ED in relation to SE compared with the suburban participants (Fig. S2). Yet there were similar levels of SE (PSS-10 subconstruct) in both cohorts and across race/ethnicity (Table 4, Fig. 1).

### PS, ED and SE are associated with the gut microbiota relative abundance but not with immune factors

**Fig. 2:**
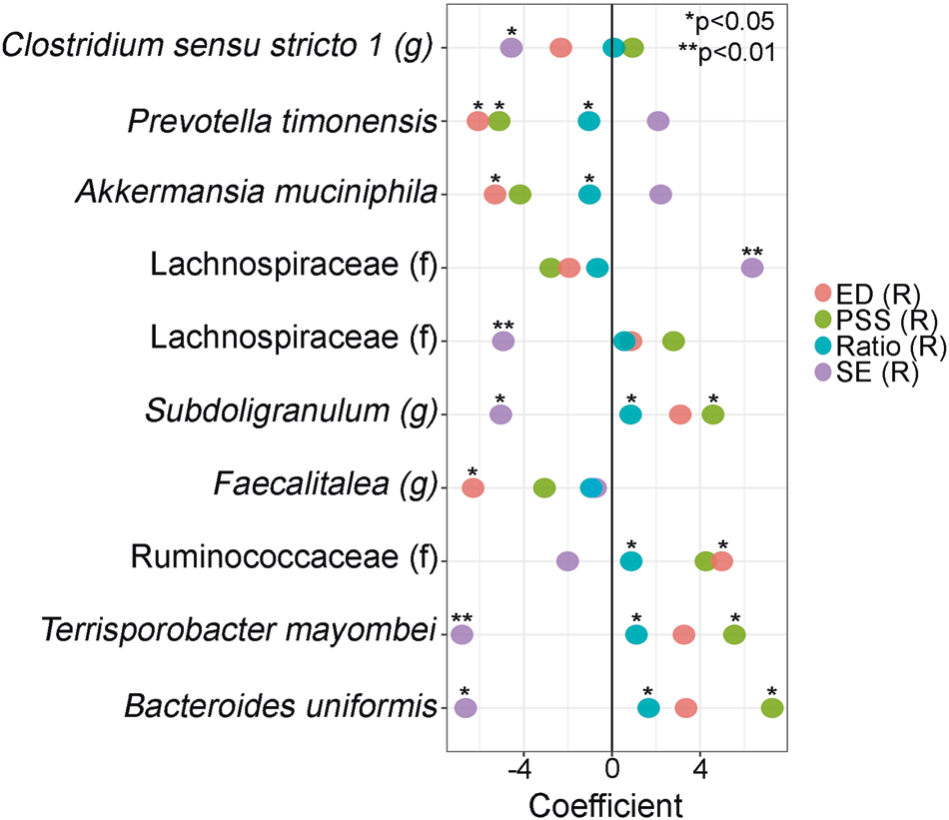
Associations between ASV with rPS, rED, rSE and reduced Ratio scores. Associations between ASV normalized counts and each factor was determined using zero-inflated Generalized Linear Models that were adjusted by recruitment site and participant’s age. Models were adjusted for multiple comparisons. *q < 0.05; **q < 0.01.

While we didn’t observe any significant differences in microbial community structures (alpha- and beta-diversity and volatility) and reported levels of rPS, rED and rSE levels (Figs. S3–S7), 10 amplicon sequence variances (ASVs) were significantly associated with rPS, rED and rSE levels and with the Ratio between rED and rSE independently of the recruitment location (Fig. 2). For instance, *Bacteroides uniformis* and *Terrisporobacter mayombei* were negatively associated with rSE while positively associated with PS, and *Prevotella timonensis* was negatively linked with both rPS and rED. No ASVs were statistical associated simultaneously with both ED and SE.

**Fig. 3:**
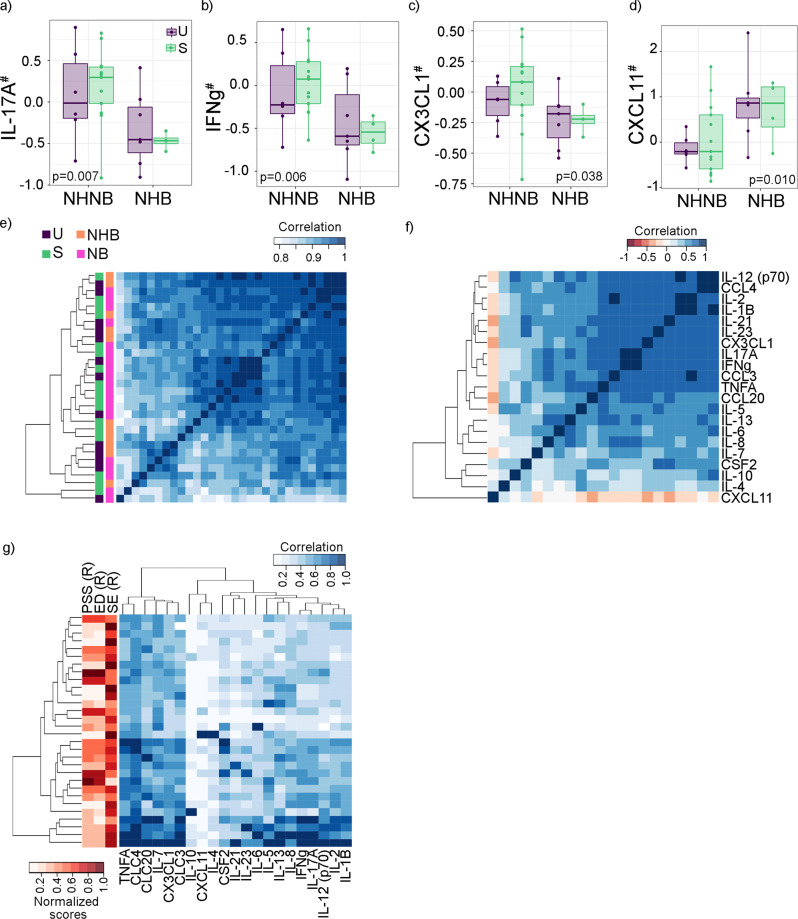
Immune system, race and rPS scores during the second trimester of gestation. **a**–**d** Several cytokines were associated with race and ethnicity—models were corrected by participants’ BMI; **e** Associations between cytokines and chemokine levels (log scale) corrected by participants’ BMI and recruitment site. **f** Partial Spearman correlation between cytokines and chemokines levels in serum at the second trimester correcting for participants’ BMI. While most of the cytokines and chemokines positively correlated with each other, only CCXL11 concentrations in serum were negatively correlated with the rest. **g** Clustering of cytokines and chemokine (log normalized) level by participant.

**Fig. 4:**
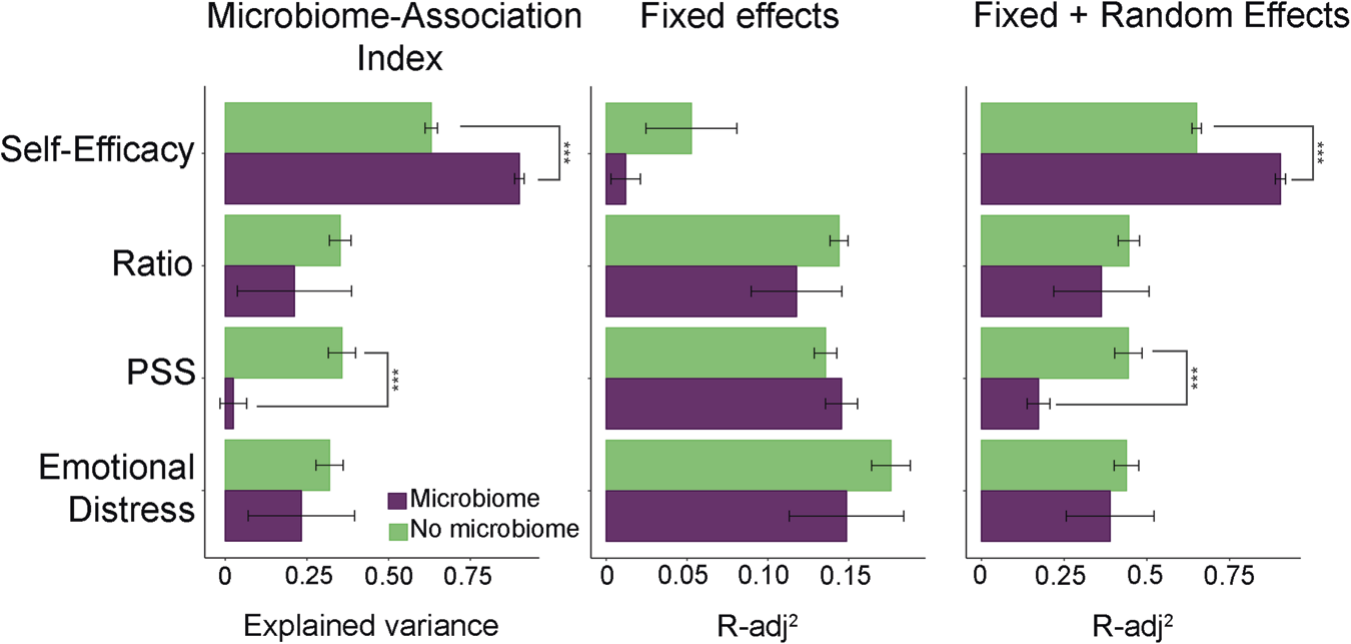
Microbiota structure predictability of self-reported PS levels using Linear Mixed models. **a** Variability of rPS, rED and rSE explained by the gut microbiota composition; **b** By socio-demographic values; **c** Maximum explained variability by the Linear Mixed Models. Fixed factors were race/ethnicity for ED, PS and Ratio and nulliparity for SE. ***q < 0.001.

There were some differences in cytokines and chemokines based on race/ethnicity (Fig. 3a–d), but none were associated with the PS factors (*p* > 0.05). Most cytokines and chemokines were positively correlated with each other, except CXCL11, which was negatively associated with several immune markers including CX3CL1, TNFa, IL-5 and CCL3. The cytokines and chemokines pairs that were highly associated with each other, included IL-1b and IL-2, IL-23 and IL-21 and IL-17a and IFN-gamma (Fig. 4f, rho > 0.9, *q* < 0.001). Fig. 3a–d include subjects from the urban cohort (purple) and subjects from the suburban cohort (green); NHNB is Non-Hispanic Non-Black and NHB is Non-Hispanic Black.

When exploring the associations between the maternal cytokine and chemokine levels in serum and the maternal gut microbiome in the second trimester, three immune markers, CXCL11, IL-5 and TNFa, were associated with seven ASVs after correcting by BMI, age and recruitment site (*q* < 0.005, Table 5). CXCL11 was negatively associated with *Bacteroides uniformis* and unidentified members of the family *Ruminococcaceae* genus *UCG-002* and of the family *Lachnospiraceae*. IL-5 was negatively associated with the genus *Methanobrevibacter* of Archaea (a methane producer) and TNFa was also negatively associated with members of the family *Ruminococcaceae* genus *UCG-002* and *Coprococcus-2*. Both IL-5 and TNFa were positively correlated with an ASV mapped to an unidentified member of the genus *Enterococcus* (Table 5).

**Table 5:** Significant associations between cytokines levels and ASV composition during the second trimester.

Cytokines (pg/ml)	Taxa	Coefficient	*q*
CXCL11	f_Lachnospiraceae	−0.02	5.E−03
CXCL11	*Bacteroides_uniformis*	−0.02	5.E−03
CXCL11	*g_Ruminococcaceae_UCG-002*	−0.03	5.E−03
IL5	*g_Enterococcus*	2.53	3.E−03
IL5	*g_Methanobrevibacter*	−1.67	4.E−03
TNFA	*g_Enterococcus*	2.36	1.E−03
TNFA	*g_Ruminococcaceae_UCG-002*	−1.10	1.E−03
TNFA	*g_Coprococcus_2*	−1.33	1.E−03

Zero-inflated Generalized Linear Models were corrected by BMI, participant age as both altered inflammatory levels-- and recruitment site, as an indirect measure of other factors that can alter inflammatory levels, such as diet.

### Predictive capabilities of the gut microbiota to explain the variations in dimensionality scores during pregnancy

Linear Mixed Models (LMM) were employed to estimate the predictive capability of the maternal gut microbiota for the self-reported scores of the rPS, rED and rSE and compared them with the predictive capabilities of LMMs that included only socio-demographic factors (Fig. 4a–c, each reflecting the different models). More than 80% of the observed variability in the rSE factor could be explained by the inclusion of the gut microbiome association index (*p* value < 0.001), significantly increasing the prediction capabilities of the LMMs by more than 25%. We didn’t observe a significant improvement in the prediction of rED or rPS levels.

### *B. uniformis* as a potential mediator between PS and immune system during pregnancy

**Fig. 5:**
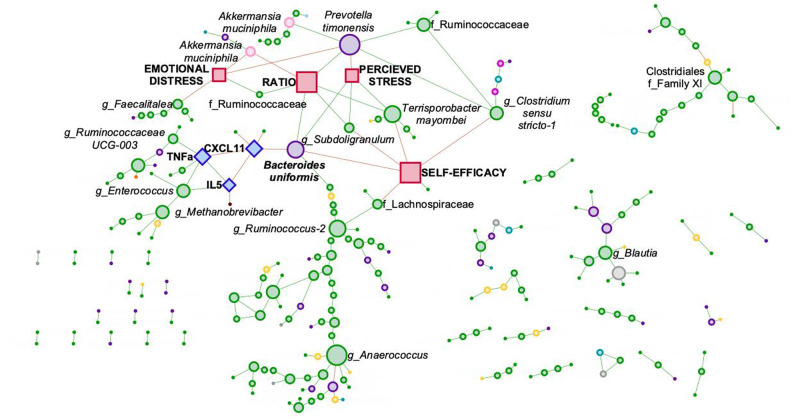
*Bacteroides uniformis* mediates the associations between the immune system, through CCXL11, and the self-reported rPS assessments, through rSE and the ratio between rED and rSE. Associations between cytokines and chemokines with correlation coefficients were >0.65 and *q* < 0.001 were included in the figure. Associations with rPS, rED, rSE and rRatio were considered significance with a *q* < 0.05. Associations between ASV were calculated with SparCc. Node size is associated with its number of neighbors. ASVs, circles; cytokines, diamonds; stress scores, squares. Firmicutes, green; Bacteroidetes, purple; Actinobacteria, yellow; unidentified bacteria, gray; Verrucomicrobia, light pink; Protobacteria, blue; Euryarchaeota, brown; Epsilonbactereota, orange; Tenericutes, light blue; Fusobacteria, fuchsia. Green edges, positive correlations; red, negative correlations.

Merging co-abundance correlations between the gut microbiota, immune system, and levels of PS rendered multiple networks; with one of the networks containing more than 95% of the significant ASVs (Fig. 5). Within this larger network, *B. uniformis* interconnected three modules or communities characterized by immune markers, factors of rPS and ASVs. *B. uniformis* was negatively associated with the chemokine CXCL11 and rSE, while positively associated with rPS and the ratio between rED and rSE. Although we didn’t identify any statistically significant associations between the immune system markers and the self-reported levels of PS, *B. uniformis* mediated the associations between the two systems, such that CXCL11 was positively associated with rSE scores and inversely correlated with rPS scored (note that the opposite is true for IL-5 and TNFa). The levels of rED were negatively correlated with several microbial ASVs such as *Prevotella timonensis*, *Akkermansia muciniphila*, and a member of the genus *Faecalitalea*; but not directly to any of the measured immune markers.

## Discussion

Perceived Stress (PS) is a complex construct that results from the balance of the individual’s emotional sense of distress (ED) and their coping mechanisms (SE). The microbiota-gut-brain axis including the host immune responses may reflect PS. Our results identified links between distinct taxonomical and immunological signatures and PS, ED and SE during mid and late pregnancy, improving predictive capability of SE in two distinctly different cohorts in terms of socio-demographics.

To assess PS similarly in both cohorts, in relation to microbial composition and the host immune system, we first determined that the two factors of PS, ED and SE, differed due to several questions not being consistent between the urban and the suburban cohort. The three questions that varied between the two cohorts related to the impact of external forces as stressors (PSS-10, item f) and the individual’s sense of their ability to manage these stressors (PSS-10, items e and h). Similar to our findings, Santiago, et al. found that a two-factor structure of PS of ED and SE was not consistent in a group of Aboriginal Australians [39]; who, like American Blacks, face structural inequalities and racism. The data-driven Woods-Giscombe’s Superwoman schema is helpful in interpreting our results [12]. The literature is mixed on the role of education in relation to perinatal anxiety [56]. It might be plausible that the greater self-reported ED levels in the suburban cohort, who were primarily Caucasian and not recruited directly from clinic may have allowed these participants to freely express their ED without fear of repercussions (e.g., worry that reporting distress will be viewed as not capable of parenting). It is also plausible that structural sexism at work might have been a contributor to greater levels of ED [57, 58]. While individuals in the suburban cohort were more likely to be partnered, the quality of their relationships was not measured—quality of relationships for those in the urban cohort were not assessed either. These findings highlight the complexity of PS, particularly in identifying those most at risk of complications such as postpartum depression or anxiety, and how individuals express PS may be impacted by culture and environment.

The host immune system reflects genetic and other factors that in bidirectional interactions with the gut microbiome may alter the effects of PS and be altered by PS. Our results confirmed that rED levels were associated with more pathogenic and less beneficial microbial communities. For instance, lower abundance of *A. muciniphila*—inversely correlated with rED, rPS and rRatio— is associated with inadequate gut barrier function, higher levels of inflammation, insulin resistance, and higher prevalence of chronic disorders [59–65]; while higher levels of the pathogenic *T. mayombei—*inversely associated with rSE and positively associated with rPS and rRatio*—*is associated with illnesses such as diabetes [66, 67]. Multiple taxa priorly associated with cardiovascular disease and type 2 diabetes were linked with specific cytokines (e.g., greater *Enterococcus* and IL-5 and TNF-a, lower *Coprococcus* and TNF-a), possibly indicating a shift to a more inflammatory state [68, 69]. Moreover, the predictive capability of the maternal gut microbial footprint during pregnancy to identify levels of self-efficacy was significantly better than models that just employed socio-demographic characteristics alone (e.g., age, marital status). These results underscore that the perception of the subject’s environmental stressors could regulate or be regulated by the maternal gut microbiota.

Our data indicates that the maternal gut microbiota may be a mediator between rSE levels and the immune system. *Bacteroides uniformis* negatively associated with CXCL11 and rSE and positively associated with rPS, connecting the immune factor and self-report measures. Prior studies have reported both positive and negative effects of *B. uniformis* outside pregnancy. *Bacteroides* are associated with negatively impacting the gut mucosal layer and have been found to be increased with a social disruption stressor in mice [70, 71]. Elevated levels of *B. uniformis* have been linked with depression, results that have been reproduced in animal models [72], but not in the perinatal period. *Bacteroides* species could harbor an environment inducive for facultative anaerobes in the gut to flourish and hostile for well-known probiotics, such as *Lactobacillus*. However, *B. uniformis* also metabolizes tryptophan, the precursor of serotonin; and therefore the presence of *B. uniformis* in the gut could possibly be beneficial for the host [71, 73]. In fact, as a probiotic, *B. uniformi*s has been shown to be efficacious to treat obesity and binge eating, which are immunometabolic forms of depression [74–77]. Further, our results indicated a negative association between *B. uniformi*s and CXCL11 during the second trimester. CXCL11 binds to the CXCR3 receptor, which is mostly expressed in Th1 cells, in the presence of foreign antigens [78], and has also been associated with higher risk of severe COVID, gastrointestinal inflammation and autoimmune disorders [79–81]. Later in pregnancy, a more proinflammatory and insulin sensitive state is required as part of normal progression of the pregnancy and for fetal development [82]. These apparently contradictory effects of *B. uniformi*s might just be yet another indication of the importance of biological context and the necessity of mechanistic studies.

In the second trimester, a balance of pro-inflammatory, anti-inflammatory, and autoimmunity is needed to both protect mother and fetus but also ensure tolerance of the fetus. As normal pregnancy progresses, insulin resistance is characterized by lower ratio of tryptophan to kynurenine in humans and mice and lower ratio of tryptophan to kynurenine is also associated with perinatal depression and other inflammatory disorders such as preeclampsia [83–86]. Tryptophan is a key regulator of neural plasticity which is vital to the required coping and cognitive adaptations necessary for a healthy sense of self-efficacy [87]. In the case of pregnant individuals with low SE levels, increased concentrations of *B. uniformis* might be necessary to compensate the reduction of tryptophan as pregnancy progresses. *B. uniformis* in combination with fiber intake is also associated with reduced inflammation [74] and our group has found individuals with history of anxiety may have less dietary fiber intake [88]. Taken together, it is plausible that a dysregulated microbiota-gut-brain axis due inadequate fiber intake and low levels of SE may lead to higher levels *B. uniformis* as a compensatory mechanism CXCL11 may reflect a response to a pathogenic microbiome, less tolerance of the fetus or an immune system overreaction during the second trimester, a period characterized by lower inflammation. Whether *B. uniformis* could be a helpful probiotic for certain individuals at risk by supporting immune balance and SE in pregnancy through tryptophan metabolism will require further investigation. Similarly, the relationship of *B. uniformis* to CXCL11, dietary fiber, and other commensal microbes in the community to determine causality and mechanism of action should be further explored.

### Limitations, strength, and future directions

To better understand the contributors of PS that could lead to negative health outcomes across a diverse groups of individuals, combined cohorts are needed [38], as modeled in our study, which is a strength of our study. Another strength of this study was probing not only PS, but its two factors, SE and ED. This allowed to first identify the lack of invariance between perceived stress dimensions in populations with distinct socio-demographics and cultural differences. Secondly, our more granular approach enables to conclude that the inclusion of the gastrointestinal microbiome characteristics improved prediction of SE, but not ED or total PS. This may indicate the gut microbiome is an important indicator of social factors, which aligns with prior research [32]. Our results represent associations, not causation and so larger and more densely sampled prospectively longitudinal studies with additional more objective tools (e.g., heart rate variability, hair cortisol levels [89], clinician- or partner-rated behavioral symptoms) are needed to identify temporal causality and confirm possible mediators. In addition, a more thorough phenotyping that is utilized at multiple sites (e.g., diagnosis by mental health professionals, same diet assessment tools, social support measures, emotional regulation activities, obstetrical outcomes) will increase our understanding of consequences of the dysregulation of the microbiota-gut-brain axis during pregnancy. For instance, the lack of quantification of dietary fiber (a well-known regulator of the gut microbial communities) prevented us to determine its plausible mediation effect between self-efficacy and gut microbiota and immune system characteristics. ED is associated with Research Domain Criteria arousal constructs (arousal and regulatory systems domain) and SE with the self-knowledge subconstruct (social processes domain) [90, 91]. Self-knowledge and arousal relate with very distinct brain circuits, and in our study, SE and ED with distinct microbial communities. Future work should consider adding assessment of brain circuitry activity, especially in understanding plasticity across the perinatal period.

## Conclusion

In conclusion, our study highlights the advantages of using tools and analytic methods that characterize the interaction of the maternal gut microbiome and the immune system, the nuanced view of PS (e.g., two factor analysis and invariance testing) and the importance of considering population differences in reporting PS. Our approach also resulted in identifying a role for *B. uniformis* in the regulation of the perinatal microbiome-gut-brain axis that reflects how the microbiome may improve our understanding of the immune activation and suppression balance that must occur in responding to stressors. An approach that integrates multiple sources of information, e.g., perception, immune and microbial attributes, is critical for assessing and identifying problematic stress during the critical perinatal period; improving our ability to identify individuals at risk, guiding intervention, and minimizing the negative long-term consequences for the mother and the infant.

## Supplementary information

Supplemental Figure Descriptions

Supplemental Data

Supplemental Figure 1

Supplemental Figure 2

Supplemental Figure 3

Supplemental Figure 4

Supplemental Figure 5

Supplemental Figure 6

Supplemental Figure 7

## Data Availability

Raw data are deposited in SRA BioProject PRJNA667109. The samples employed for this study within this project are in the Table S3.

## Code availability

Code is available upon request.
