# Supplementary figures and images for: Interactions between perceived stress and microbial-host immune components: two demographically and geographically distinct pregnancy cohorts
Source: Transl Psychiatry. 2023 Jan 6;13:3. doi: 10.1038/s41398-022-02276-3 (PMC9822983; doi:10.1038/s41398-022-02276-3)

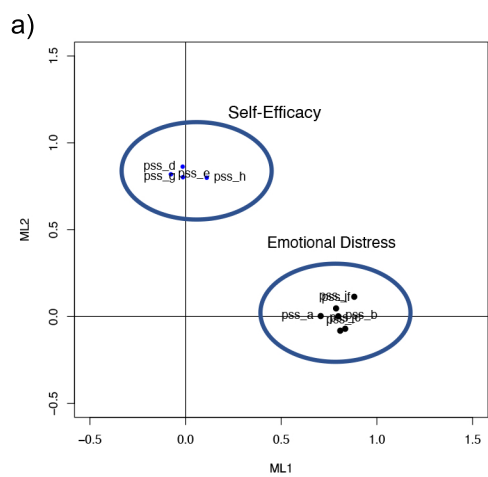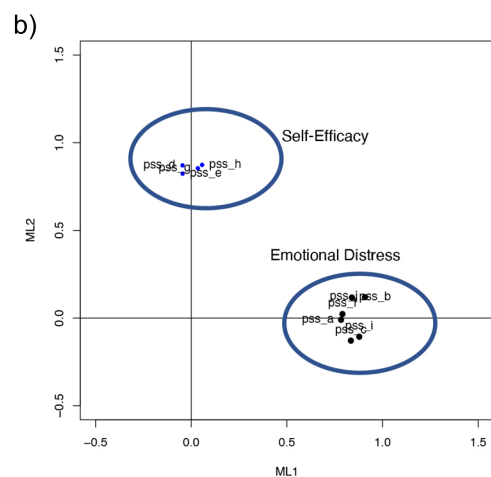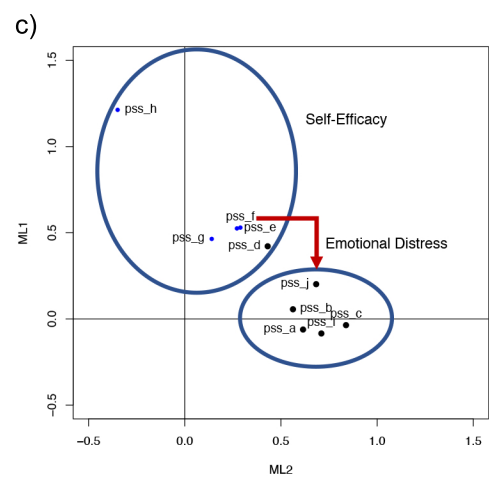

Supplement: Supplementary file 3 — Supplemental Figure 1 [file 41398_2022_2276_MOESM3_ESM.pdf]

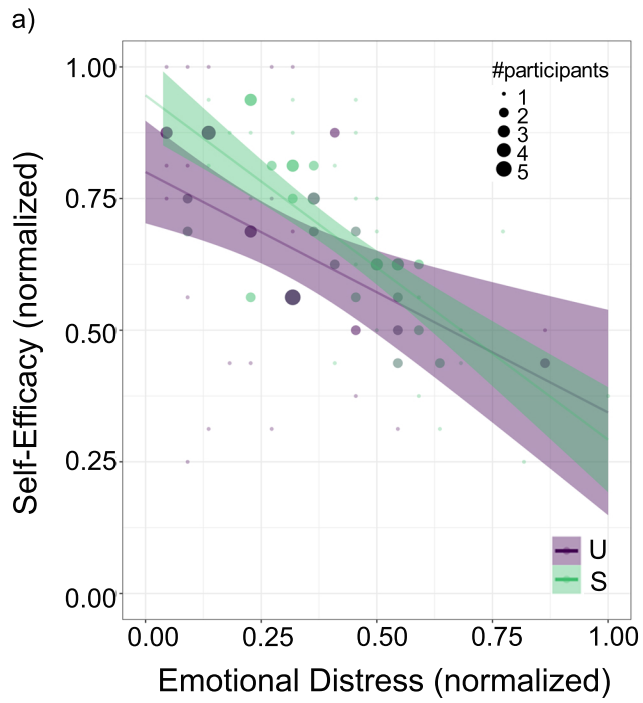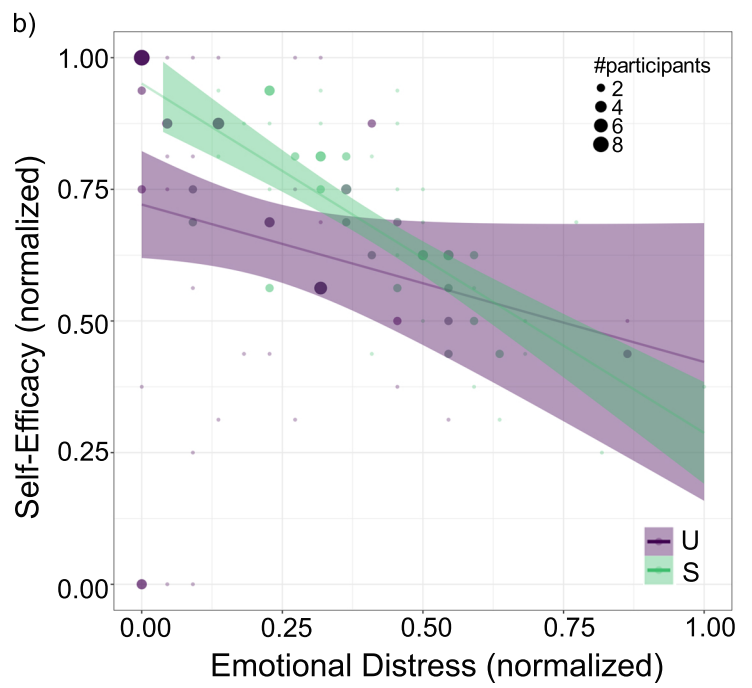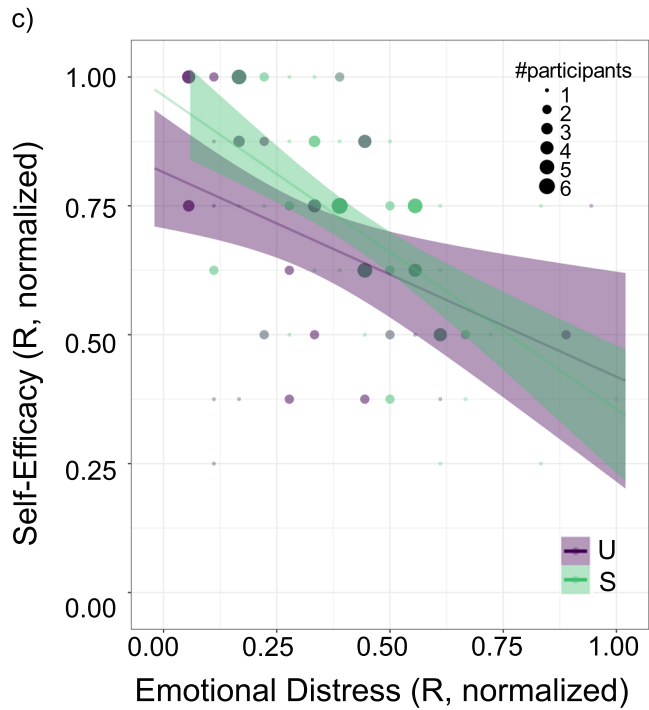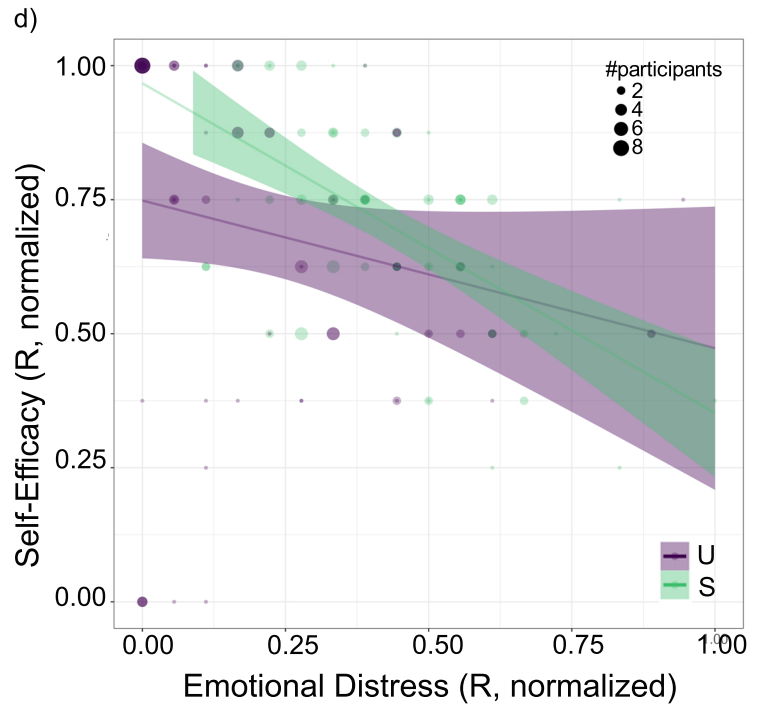

Supplement: Supplementary file 4 — Supplemental Figure 2 [file 41398_2022_2276_MOESM4_ESM.pdf]

Index

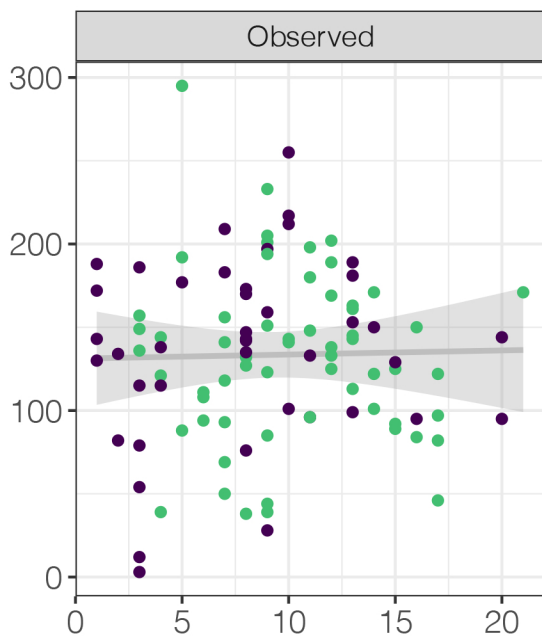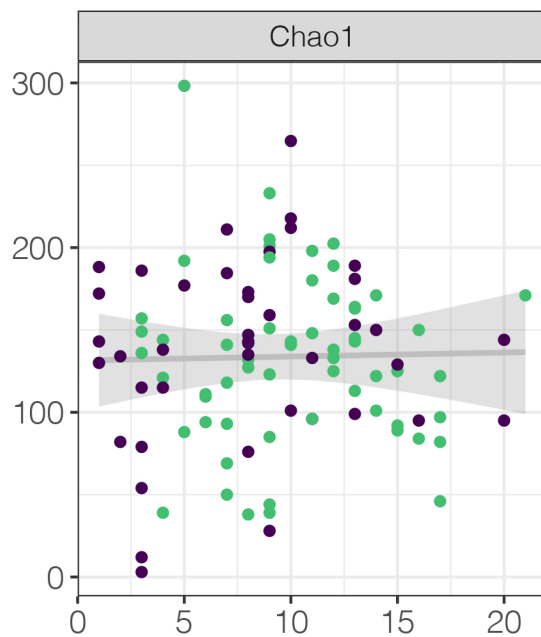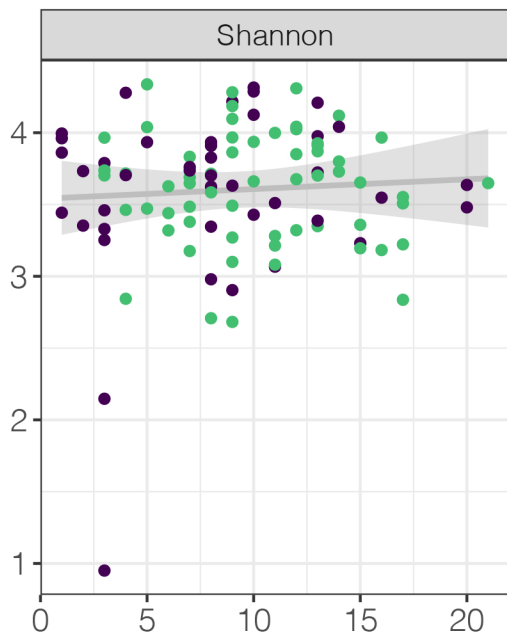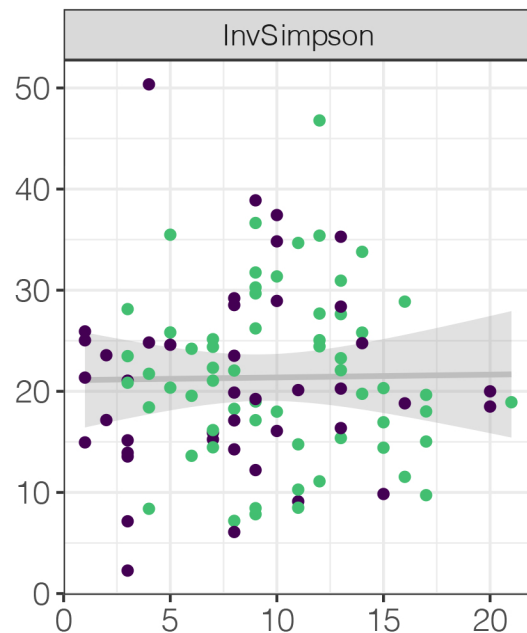

U  
S

Perceived Stress (reduced)

Supplement: Supplementary file 5 — Supplemental Figure 3 [file 41398_2022_2276_MOESM5_ESM.pdf]

Index

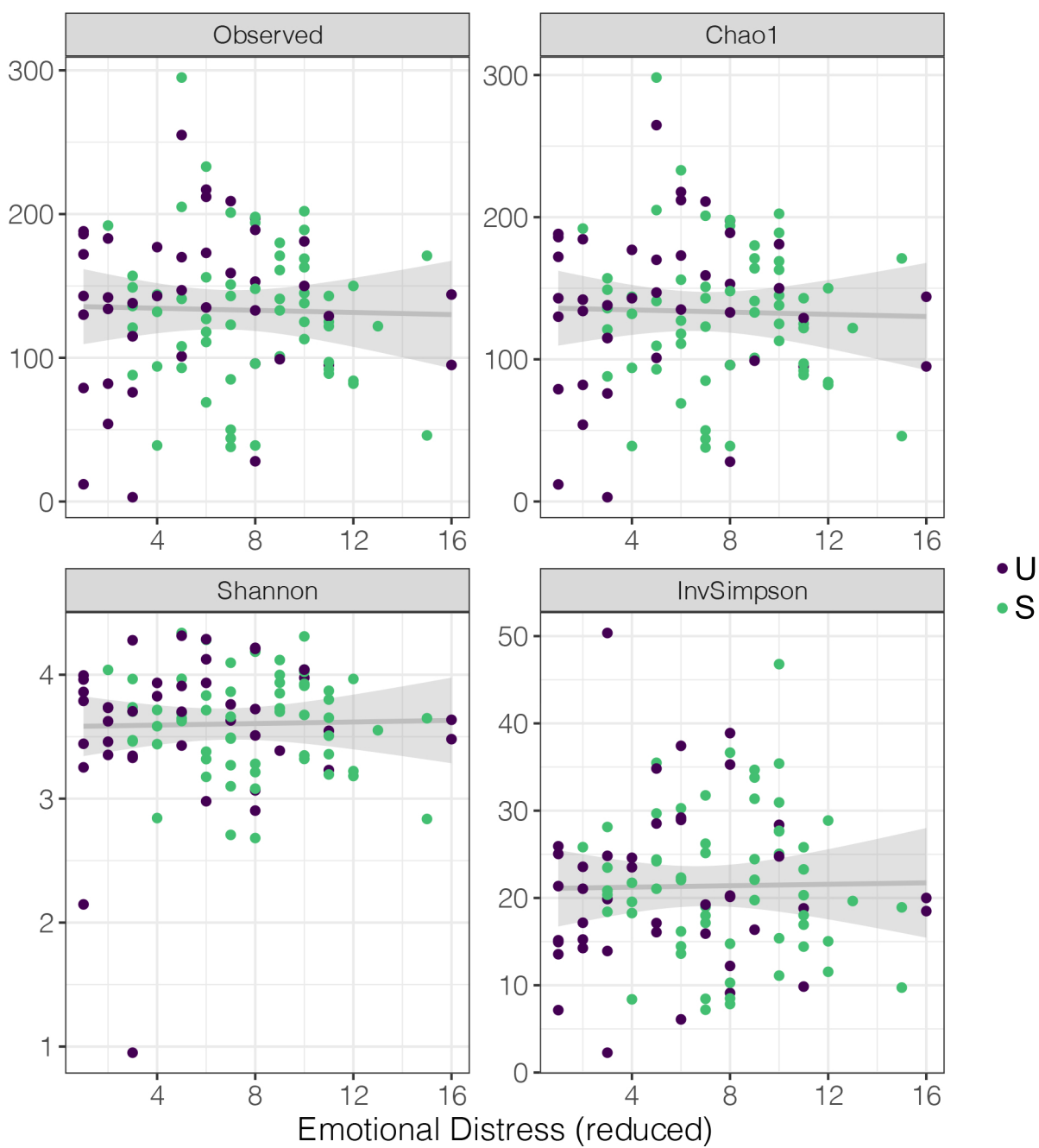

Supplement: Supplementary file 6 — Supplemental Figure 4 [file 41398_2022_2276_MOESM6_ESM.pdf]

Index

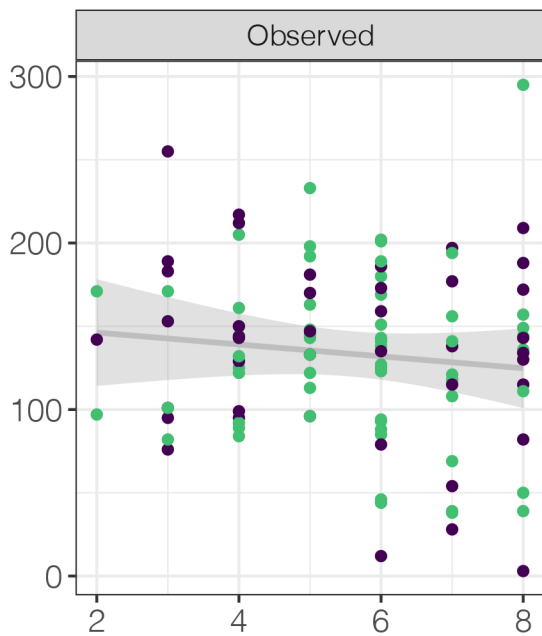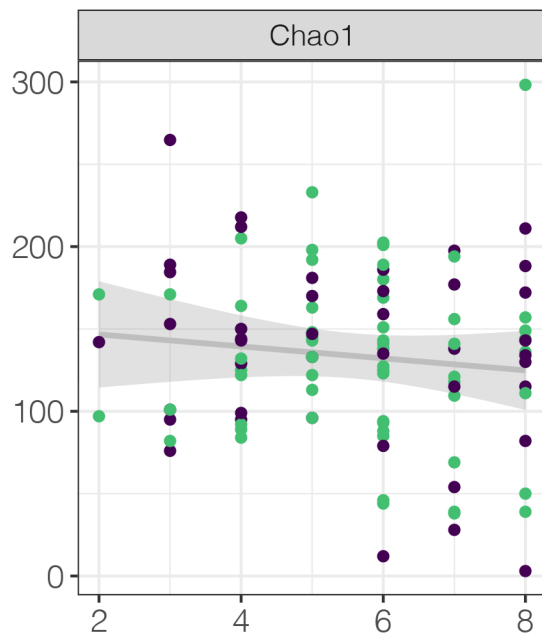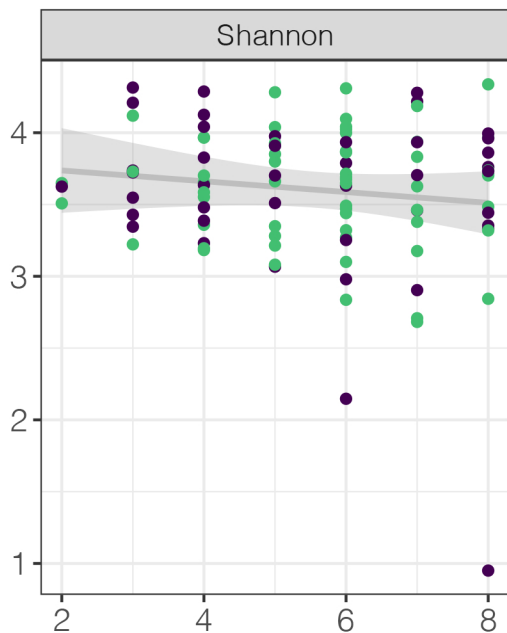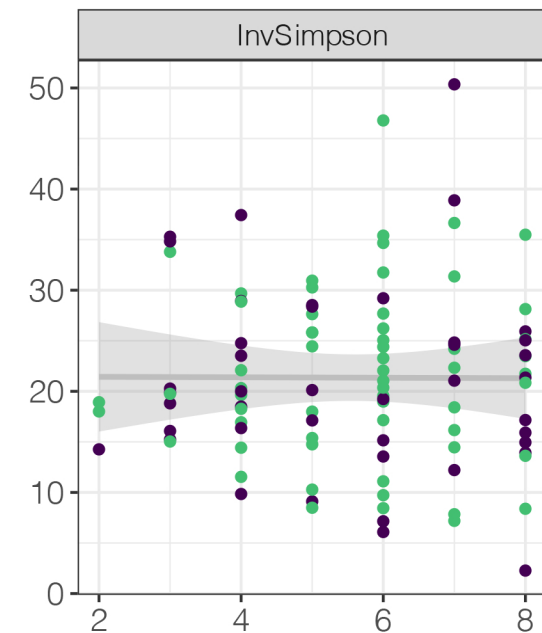

U  
S

Self-Efficacy (reduced)

Supplement: Supplementary file 7 — Supplemental Figure 5 [file 41398_2022_2276_MOESM7_ESM.pdf]

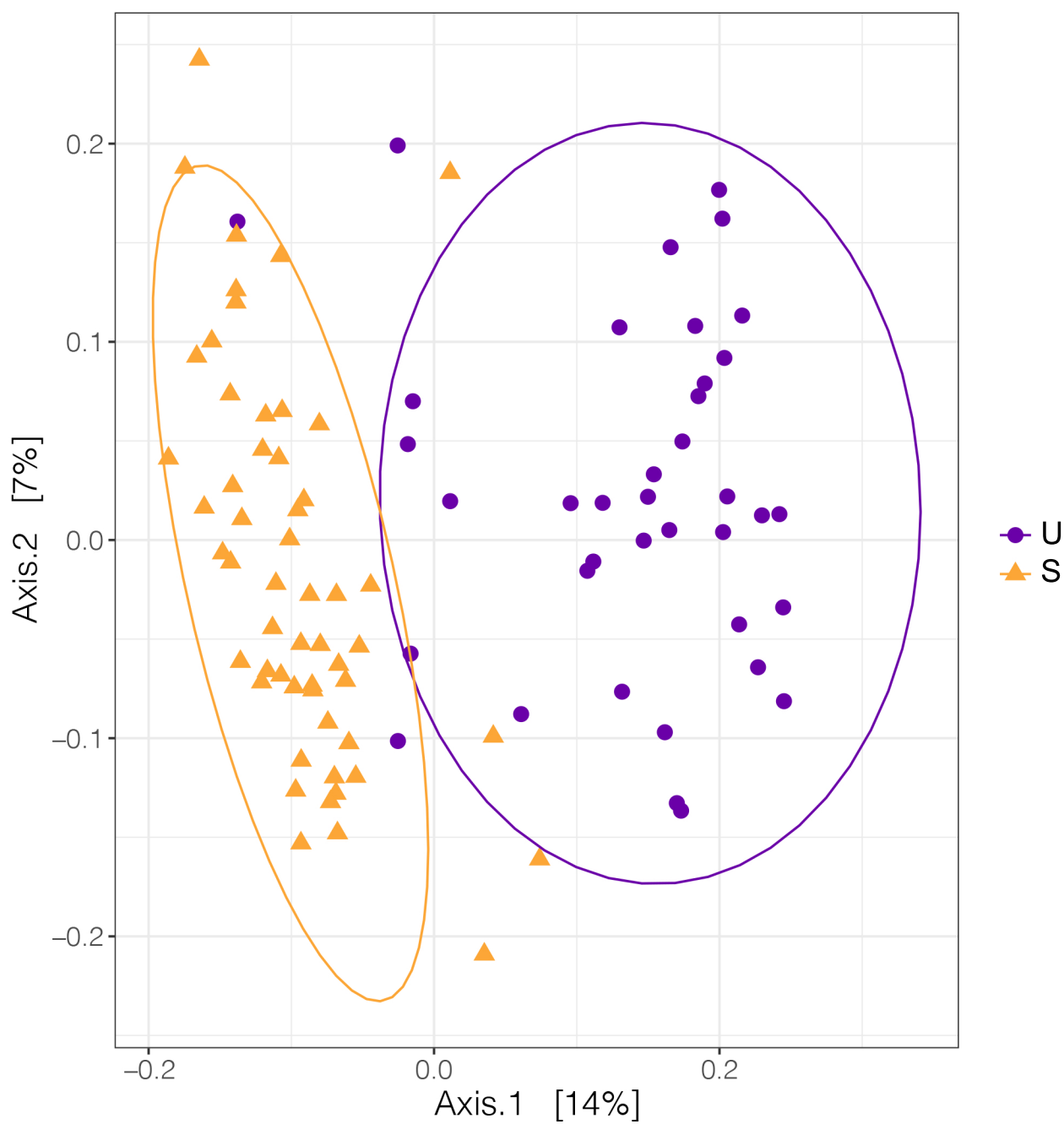

Supplement: Supplementary file 8 — Supplemental Figure 6 [file 41398_2022_2276_MOESM8_ESM.pdf]

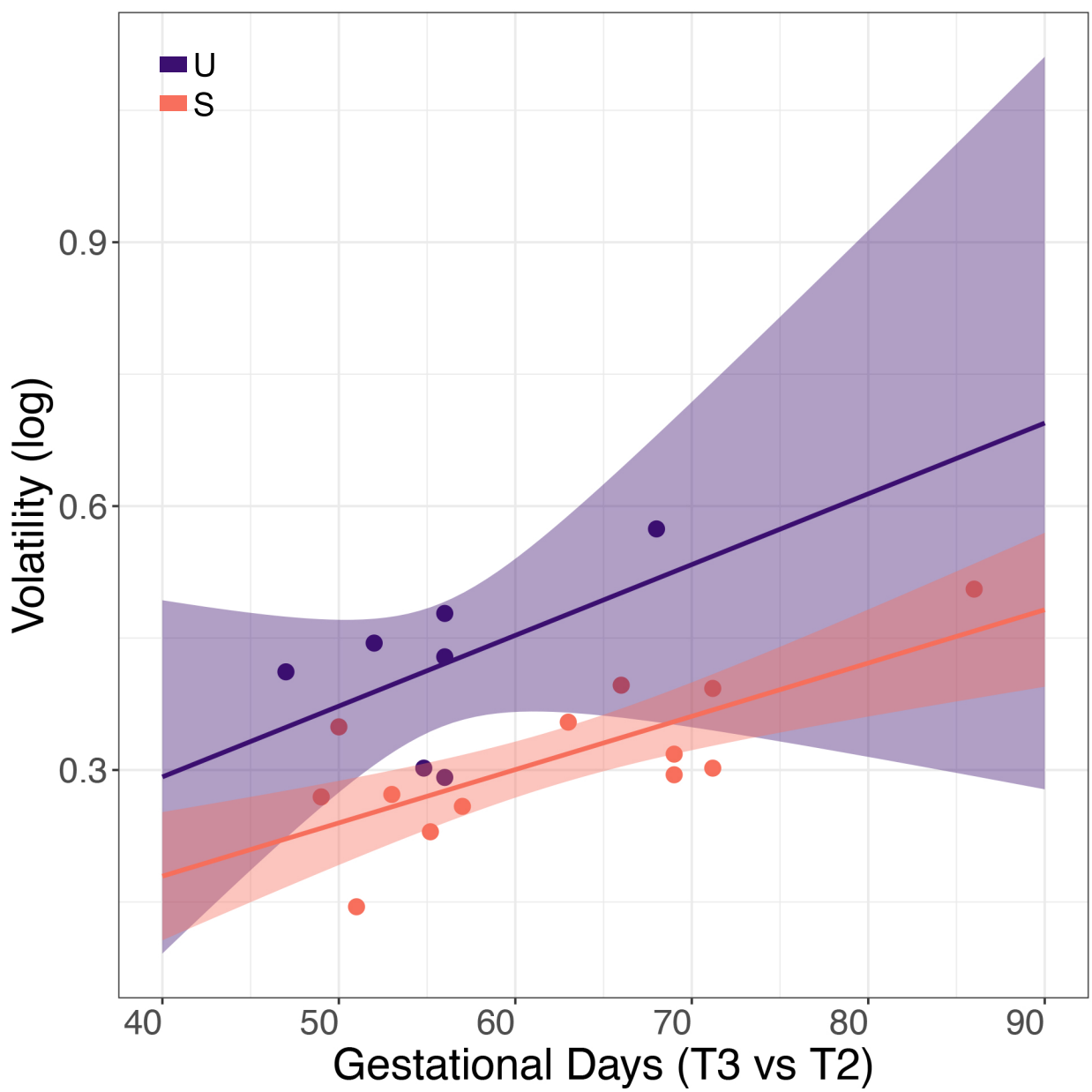

Supplement: Supplementary file 9 — Supplemental Figure 7 [file 41398_2022_2276_MOESM9_ESM.pdf]
